# Bone-filling mesh container versus percutaneous kyphoplasty in treating Kümmell’s disease

**DOI:** 10.1007/s11657-019-0656-4

**Published:** 2019-11-18

**Authors:** Z.-K. Duan, J.-F. Zou, X.-L. He, C.-D. Huang, C.-J. He

**Affiliations:** Department of Pain, Guizhou Provincial People’s Hospital, Zhongshan Road, Guiyang, 550002 Guizhou China

**Keywords:** Vertebroplasty, Kyphoplasty, Cementoplasty, Bone cements, Osteoporosis, Kümmell disease, Bone-filling mesh container

## Abstract

***Summary*:**

Kümmell’s disease (eponymous name for osteonecrosis and collapse of a vertebral body due to ischemia and non-union of anterior vertebral body wedge fractures after major trauma) cannot heal spontaneously. Bone-filling mesh container (BFMC) can significantly relieve pain, help the correction of kyphosis, and may prevent cement leakage. This pilot study may provide the basis for the design of future studies.

**Purpose:**

To compare the effectiveness and safety of BFMC and percutaneous kyphoplasty (PKP) for treatment of Kümmell’s disease.

**Methods:**

From August 2016 to May 2018, 40 patients with Kümmell’s disease were admitted to Guizhou Provincial People’s Hospital. Among them, 20 patients (20 vertebral bodies) received PKP (PKP group) and the other 20 received BFMC (BFMC group). Operation time, Visual Analogue Scale (VAS), Oswestry Disability Index (ODI), Cobb’s angle changes, and related complications were recorded.

**Results:**

All patients underwent operations successfully. VAS scores and ODI of both groups at each postoperative time point were lower than preoperatively, with statistically significant difference (*p* < 0.05). Postoperative Cobb’s angle of both groups postoperatively was lower than preoperatively (*p* < 0.05). Cement leakage occurred in eight vertebrae (8/20) in the PKP group and in one vertebra (1/20) in the BFMC group. No complications such as pulmonary embolism, paraplegia, or perioperative death occurred during operation in both groups. Adjacent vertebral refractures occurred in five patients (5/20) in the PKP group and in four patients (4/20) in the BFMC group, with no significant difference in the incidence rate of refractures in both groups but the material is too small to verify statistically.

**Conclusions:**

Both PKP and BFMC technologies can significantly relieve pain and help the correction of kyphosis while treating Kümmell’s disease. Moreover, the BMFC may prevent cement leakage.

## Introduction

Osteoporotic vertebral compressive fractures are common lesion in the elderly [1, 2]. Most patients with osteoporotic vertebral compressive fractures can have symptoms progressively relieved after several weeks of non-surgical treatment, but about 1/3 of the patients will have obvious low back pain and kyphosis [3], which may develop into Kümmell’s disease, also known as delayed vertebral collapse due to post-traumatic osteonecrosis [4]. These lesions cannot heal spontaneously. In order to relieve pain and prevent continuous vertebral collapse and aggravation of kyphosis, non-surgical and surgical treatment may be applied. However, as non-surgical treatment has poor effect, surgical treatment is often preferred [5].

Patients with Kümmell’s disease are often multi-diseased and are in poor physical condition, and crevasse filling with bone cement may theoretically stabilize the spine and obviously relive pain. For such patients, the most commonly used methods at present are percutaneous vertebroplasty (PVP) and percutaneous kyphoplasty (PKP) [6]. However, PVP and PKP often have serious complications, of which cement leakage is the most common with incidence rates of 22–82% [7]. In order to reduce the occurrence of cement leakage, bone-filling mesh container (BMFC) has been used in vertebroplasty [8]. For patients with Kümmell’s disease phase III with spinal canal compression but without neurological symptoms [9], it is not clear whether vertebroplasty is the preferable method [10]. Perusal of the literature has not revealed any report comparing the curative effect of PKP and BFMC in treating Kümmell’s disease. The purpose of the current study was to compare the effectiveness of PKP and BFMC in treatment of 40 patients with Kümmell’s disease admitted into Guizhou Provincial People’s Hospital between August 2016 and May 2018.

## Materials and methods

### General data

Inclusion criteria are as follows: (1) single vertebral Kümmell’s disease without neurological symptoms; (2) patients aged above 60 years; (3) severe osteoporosis (bone mineral density T < − 3.0 SD); (4) radiological imaging examination showing vacuum sign, fissure sign, or sclerosis at the fractures site. Exclusion criteria are as follows: (1) need decompression to relieve nerve compression; (2) multiple-level compression fractures; (3) history of vertebroplasty or spinal surgery; (4) vertebral fractures or vertebral space occupying lesion due to tuberculosis, tumor, or otherwise; (5) coagulation dysfunction; (6) surgery not tolerable due to severe internal medicine diseases.

Forty patients with Kümmell’s disease were included between August 2016 and May 2018, 22 patients with thoracic vertebrae, and 18 with lumbar vertebrae lesions. The patients were randomly divided into 2 groups, each with 20 patients. The vertebral body distribution and other general conditions are shown in Table 1. Five patients had history of hormone treatment, and 9 patients had diabetes. There were no significant statistical differences in age, gender, and vertebral body distribution between the two groups. The study was approved by the Medical Ethics Committee of Guizhou Provincial People’s Hospital.

**Table 1:** General conditions of patients in both groups

Groups	Cases	Sex ratio	*T*_11_	*T*_12_	*L*_1_	*L*_2_	*L*_3_	Phase		
		(M/F)						I	II	III
PKP group	20	8/12	4	8	5	2	1	5	10	5
BFMC group	20	9/11	0	9	5	4	2	4	10	6

### Operation methods

All patients were taken in prone position. The skin of the surgical site was disinfected and draped routinely. One percent lidocaine was given as local anesthesia, and the puncture needle was inserted into the vertebral posterior margin of the vertebral pedicle. The balloon was embedded into the anterior 1/3 of the vertebral body by solid vertebral drill for dilatation. After the balloon was withdrawn, polymethyl methacrylate bone cement was injected with a bone cement injector guided by a C-arm (Artis zeego digital subtraction angiography DSA system, Germany) in the PKP group. However, in the BFMC group, after the balloon was withdrawn, BMFC (Shandong Guanlong Medical Products Co., Ltd.) and special kyphoplasty equipment (including a puncture device and inflatable balloon) were inserted. The material of the bag body is 75D/36F high-strength wire comprising 100% polyethylene terephthalate with a fineness of 166.5 dtex and a strength of 6.75 CN/dtex. The single-layer bag is made of a relatively thick high-strength wire that exhibits good biocompatibility [8]. Polymethyl methacrylate bone cement was injected with a screw propeller with aid by the C-arm.

Patients returned to the wards without symptoms from lower limbs or respiratory discomfort after operation and were required to stay in bed for 6 h.

### Observational indices and assessment methods

Operation time, Visual Analogue Scale (VAS) [11], and Oswestry Disability Index (ODI) [12, 13] of the two groups were recorded the day before (T1) and after (T2) operation as well as 1 (T3) and 6 (T4) months after operation. Descending height and Cobb’s angle of vertebral bodies were measured with lateral X-rays. Cobb’s angle was measured through the intersection angle of the extension lines for superior endplate at the cranial vertebral body of the treated vertebra as well as for the caudal endplate at the lower vertebral body. Outpatient follow-up was performed 1 and 6 months after discharge with conventional radiography and with CT or MRI examination when considered necessary.

Cement leakage, incidence rate of cement leakage, and leakage locations could be observed through intraoperative C-arm and postoperative CT reexamination (phase I was cement leakage into spinal canal, phase II was cement leakage into paravertebral veins, phase III was cement leakage into paravertebral soft tissue, and phase IV was cement leakage into adjacent intervertebral disk) [14, 15]. Complications such as pulmonary embolism, paraplegia, and perioperative death during operation were looked for in both groups.

### Statistical analysis

Measurement data were expressed as mean ± standard deviation, while non-normally distributed data were expressed as median and quartile. Preoperative and postoperative ODI, kyphotic angle, and operation-related complications were statistically analyzed and evaluated by using repeated measure analysis of variance. VAS was ranked data, which was verified by adopting rank sum. Value *F* represented significant test statistics of results mean difference at different times, while *p* < 0.05 meant that differences were considered statistically significant.

## Results

The operation time of the PKP group was 33–55 min ([43.30 ± 7.20] min) and 32–55 min ([43.80 ± 7.61] min) in the BFMC group, with no statistical significance between the two groups. The VAS score of the PKP group at day one before and after operation as well as at 1 and 6 months after operation were 7 points (7–8 points), 2 points (2–3 points), 2 points (2–3 points), and 1 point (1–2 points), respectively, while those of the BFMC group were 7.5 points (7–8 points), 2 points (2–3 points), 1 point (1–2 points), and 1.5 points (1–2 points), respectively. VAS scores of both groups at each postoperative time point were lower than preoperatively, with statistically significant difference (*p* < 0.05, Table 2).

**Table 2:** Preoperative and postoperative VAS scores [M(Q1~Q3)]

Groups	Cases	T1	T2	T3	T4
PKP group	20	7 (7~8)	2 (2~3)a	2 (2~3)a	1 (1~2)a
BFMC group	20	7.5 (7~8)	2 (2~3)a	1 (1~2)a	1.5 (1~2)a
*Z* value		− 0.73	− 0.44	− 1.58	− 0.62
*p* value		0.94	0.66	0.11	0.53

Note: Compared with T1, *p* < 0.05a was considered to indicate a significant difference

*T1*, before operation; *T2*, after operation; *T3*, 1 month; *T4*, 6 months

The ODI values of at day one before and after operation as well as at 1 and 6 months after operation were 75.50 ± 4.48, 26.75 ± 2.81, 16.80 ± 1.44, and 12.75 ± 1.29, respectively, while those of the BFMC group were 75.45 ± 4.34, 26.40 ± 2.35, 15.95 ± 1.10, and 11.95 ± 1.15, respectively. ODI scores of both groups at each postoperative time point were lower than those at preoperative time point, and the difference was statistically significant (*p* < 0.05, Table 3).

**Table 3:** Comparison of preoperative and postoperative ODI values

Groups	T1	T2	T3	T4	*F* value	*p* value
PKP group	75.50 ± 4.48	26.75 ± 2.81a	16.80 ± 1.44a	12.75 ± 1.29a	2130.69	0.00
BFMC group	75.45 ± 4.34	26.40 ± 2.35a	15.95 ± 1.10a	11.95 ± 1.15a	2387.34	0.00
*t* value	0.02	0.68	1.39	0.90		
*p* value	0.89	0.42	0.25	0.35		

Compared with T1, *p* < 0.05a was considered to indicate a significant difference

*T1*, before operation; *T2*, after operation; *T3*, 1 month; *T4*, 6 months

Cobb’s angle of the PKP group before and after operation was (22.90 ± 0.96)° and (16.90 ± 1.37)°, respectively, while that of the BFMC group was (23.16 ± 0.83)° and (16.79 ± 1.59)°, respectively. Values of postoperative Cobb’s angle of both groups were lower than those of preoperative Cobb’s angle, with statistically significant difference (*p* < 0.05, Table 4).

**Table 4:** Comparison of preoperative and postoperative Cobb’s angle between the two groups

Groups	Pre-operation	Post-operation	*F* value	*p* value
PKP group	22.90 ± 0.96	16.90 ± 1.37a	16.35	0.00
BFMC group	23.16 ± 0.83	16.79 ± 1.59a	15.85	0.00

Compared with pre-operation, *p* < 0.05a was considered to indicate a significant difference

Eight vertebrae (8/20) in the PKP group had cement leakage into the intervertebral disk and paravertebral tissues during operation, but no patient showed any clinical symptoms. Five patients had leakage into intervertebral space, two into paravertebral tissues or paravertebral veins, and one into the spinal canal. Only one vertebra (1/20) in the BFMC group had cement leakage into paravertebral tissues, with a cement leakage rate obviously lower than that of the PKP group, with a statistically significant difference (*p* < 0.05, Table 5).

**Table 5:** Bone cement leakage

Group	I	II	III	IV	Average bone cement leakage rate
PKP group	1	2	5	0	8/20
BFMC group	0	0	1	0	1/20a

Compared with PKP, *p* < 0.05a was considered to indicate a significant difference

*Type I leakage*, leaks to spinal cord; *Type II leakage*, leaks to paraspinal vein; *Type III leakage*, adjacent vertebral soft tissue; *Type IV leakage*, adjacent disk

There were no perioperative complications. Adjacent vertebral fractures occurred in 5 (5/20) patients in the PKP group and in 4 (4/20) patients in the BFMC group, which showed no statistical difference between the two groups.

## Discussions

Osteoporotic vertebral compression fractures are a common fractures in the elderly. Most patients can have symptoms relieved after several weeks of non-surgical treatment, but about 1/3 of the patients have continuous pain and discomfort and about 10% of the patients will develop vertebral collapse due to post-traumatic osteonecrosis [16]. In 1895, Hermann Kümmell described the lesion for the first time, which is also known as Kümmell’s disease. Kümmell’s disease mainly occurs in the thoracolumbar part and is delayed vertebral collapse due to post-traumatic osteonecrosis [17].

At present, non-surgical and surgical treatments are used to treat Kümmell’s disease. Most of Kümmel’s diseases often develop into chronic back pain or even cripple [5]. Surgical treatment can quickly relieve patient’s pain [18], correct kyphosis, and reduce complications due to long-term bed rest and is therefore widely used. Surgical treatment mainly includes PVP, PKP, and open anterior and posterior approach operations [19, 20]. Patients with Kümmell’s disease are mostly elderly who are in poor general condition, and most of whom are multi-diseased, with poor tolerance to operation, and suffer high risk of open internal with more complications. Because of severe osteoporosis, even when the fixation is carried out with pedicle screw augmentation technique, complications [21] such as internal fixation failure and low fusion rate are common. Patients with Kümmell’s disease in phase III are especially exposed to have complications; thus, PVP and PKP are often used in clinical practice. Cement leakage is the most common complication of PVP with a leakage rate of 22–82% [22, 23]^.^ However, in previous studies, it has been reported that PKP has advantages over PVP in reducing the cement leakage rate [24–27]. In order to further reduce the occurrence of cement leakage, BMFC technology is now introduced in clinical practice. In a report, He [8] found that BFMC can effectively prevent bone cement leakage and reduce the incidence of bone cement leakage.

In our study, VAS scores of patients in both groups at each postoperative time point are all lower than preoperative ones (*p* < 0.05). Oswestry Disability Indexes (ODI) at each postoperative time point are all lower than preoperative ones, and the differences are statistically significant (*p* < 0.05). Postoperative Cobb's angle value postoperative is lower than that preoperative,with statistical difference (*p* < 0.05), which indicates that both simple percutaneous kyphoplasty and percutaneous kyphoplasty with BMFC have good curative effects of significantly relieving pain, increasing vertebral strength, effectively preventing the aggravation of kyphosis, and obviously improving patients’ living quality.

In the current study, cement leakage was found in only one case in the BFMC group, with significantly lower leakage rate than that in PKP group, but it should be regarded cautiously due to the small sample sizes. The BFMC used in the current study is a newly developed inflatable mesh-bag-shaped bone filler that is produced by Shandong Guanlong Medical Products Co. Ltd. Shandong, China. During the cement injection of PVP augmentation, bone cement can fully diffuse through specially designed meshes, and intravertebral cement distribution can be controlled by mesh bag to reduce the risk of cement leakage.

Follow-up reveals that some patients have adjacent vertebral fractures that often occur in the inferior adjacent vertebral body of the diseased vertebra. Five patients in the PKP group have postoperative adjacent vertebral refractures, compared with 4 patients in the BFMC group, but with no statistical difference. The causes for adjacent vertebral refractures may be the high strength of polymethyl methacrylate bone cement, which generates “pillar effect” after being injected into the vertebra [28], which then easily causes degeneration of adjacent intervertebral disk and reduces the buffering effect of intervertebral disk to some extent. The abnormal stress distribution and causes obvious increase of fractures at adjacent vertebra. While others reject this notion, metanalysis and studies have shown that the incidence of adjacent fractures is similar between conservative management and PKP, so neighboring fractures are probably the natural sequela of an osteoporotic spinal fractures [29]. The solution against adjacent fractures is to perform vertebroplasty [30].

**Fig. 1:**
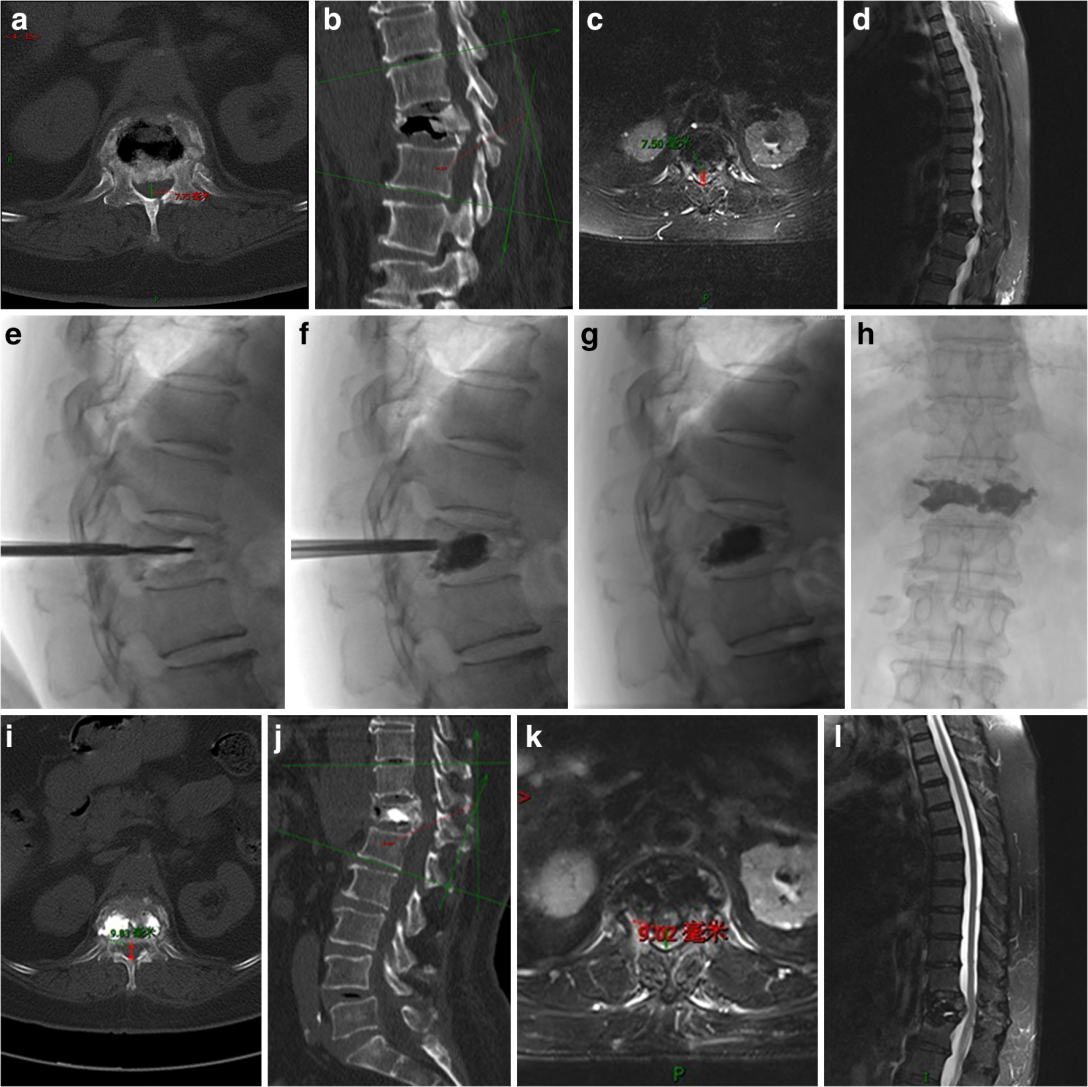
A 79-year-old female patient admitted because of chest and low back pain for more than 1 month. CT and MRI showed Kummell’s disease phase III with spinal canal compression but without neurological symptoms. Before operation (**a**–**d**): MRI and CT showed *T*_12_ level with an intravertebral vacuum cleft. Vertebral body and adnexal bone were interrupted continuously with vertebral wedge (compression 1/2). The posterior margin of the vertebral body bulges backwards, the anteroposterior diameter (7.5–7.75 mm) of the spinal canal was narrowed and Cobb’s angle was 24.22°. During operation (**e**–**h**): anteroposterior and lateral view showed the placement of the bone-filling mesh container. The vertebral body was punctured through the bilateral vertebral pedicle approach, the mesh bag implanted, and the bone cement filled well without leakage. After operation (**i**–**l**): MRI showed the T_12_ vertebra filled with bone cement. Vertebral body angle was improved and the compression reduced to 1/3. CT showed the widened diameter (9.02–9.83 mm) of the spinal canal, Cobb’s angle was 19.93°, and bone cement filled well without leakage

In our material of mainly Kümmell’s disease in phases I and II, a few have only spinal canal compression without neurological symptoms in phase III (Fig. 1). The recovery extent of vertebral height is more obvious in the BMFC than in the PKP group as the enclosing effect of BMFC makes bone cement slowly permeate in the mesh bag, reduces leakage rate, and ensures sufficient bone cement injection. Hence, the vertebra is elevated quite well.

In conclusion, while both BMFC and PKP can relieve pain, restore vertebral height, and correct kyphosis, BFMC may also reduce the incidence rate of cement leakage during operation for patients with Kümmell’s disease in phase III with posterior wall breakage and spinal canal compression but without neurological symptoms. Therefore, considering the effectiveness and safety of the treatment of Kümmell’s disease in phase III with bone cortex breakage, BMFC should be further tested.

As BMFC technology has been applied for treatment of Kümmell’s disease for a short time, this pilot study may provide the basis for the design of future studies. There are few reports about mesh bag and long-term follow-up data, and large-sampled, multi-center and long-term follow-up studies need to be conducted.
